# Tumor Deposits in Non-Metastatic Colorectal Cancer as a Risk Factor of Peritoneal Metastasis

**DOI:** 10.7150/ijms.113099

**Published:** 2025-06-23

**Authors:** Alberto Vilar Tabanera, Manuel Díez Alonso, Yousef Allaoua, Lucas Casalduero, Fernando Mendoza Moreno, Félix Mañes, Belén Matías, Lucía Diego, Cristina Vera Mansilla, Laura Castellá Bataller, Raúl Diaz-Pedrero, Miguel A Ortega, Melchor Álvarez de Mon, Alberto Gutiérrez

**Affiliations:** 1 Department of General and Digestive Surgery, University Hospital Príncipe de Asturias, 28805 Madrid, Spain.; 2 Department of Surgery, Medical and Social Sciences. Faculty of Medicine and Health Sciences, University of Alcalá, 28801 Alcalá de Henares, Spain.; 3 Department of Anatomical Pathology, University Hospital Príncipe de Asturias, 28805 Madrid, Spain.; 4 Ramón y Cajal Institute of Sanitary Research (IRYCIS), University Hospital Príncipe de Asturias, 28034 Madrid, Spain.; 5 Department of Medicine and Medical Specialities, (CIBEREHD), Faculty of Medicine and Health Sciences, University of Alcalá, 28801 Alcalá de Henares, Spain.; 6 Immune System Diseases-Rheumatology and Internal Medicine Service, University Hospital Príncipe de Asturias, (CIBEREHD), 28806 Alcalá de Henares, Spain.

**Keywords:** Tumor deposits, peritoneal metastasis, colorectal cancer.

## Abstract

**Background:** Although tumor deposits (TD) have been known for almost a century, their origin and mode of spread remain controversial. The main objective is to analyze the prognostic value of tumor deposits in non-metastatic colorectal cancer as a risk factor of global recurrence, locoregional recurrence, liver and lung metastasis and specially for peritoneal metastasis (PM).

**Methods**: This study analyzed 1,425 non-metastatic colorectal cancer patients. Four groups were built, according to the presence or absence of Lymph Node Metastasis (LNM) or TD.

**Results**: The global recurrence rate in patients with TDs was significantly higher than those without TDs (17.8% vs 60.8%; p<0.001). Patients with TDs had a lower survival and suffered higher rates of liver metastasis (8.6% vs 26.7%; p<0.001); OR of 4.244 (95% CI: 3.004-5.994) and lung metastasis (7.4% vs 19.3%; p<0.001); (OR 3.585;95% CI: 2.397-5.362). However, the main differences were found in PM (4.7 % vs 26.1 %; p<0.001); (OR: 7.511 (95% CI:5.092-11.079). Distribution by groups shows that patients with TD and LNM had a higher rate of PM. Patients with TD without any LMN had higher PM rate than those with LNM without TD. In stage III, patients with TD suffered higher rates of PM, (26.1% vs 10.9%); p< 0.001). OR: 3.075 (95% CI: 1.969-4.803).

**Conclusions**: The presence of TD increases the risk of peritoneal metastasis. Patients with TD without LNM had higher rate of peritoneal metastasis than those with LNM without TD. TD have independent prognostic value and provide complementary information. Prognostic value of TDs is underestimated in the current TNM system.

## Introduction

Colorectal cancer (CRC) is the third most frequent tumor worldwide and is one of the main causes of cancer mortality. The most common metastasis location is the liver, followed by the lung and thirdly the peritoneal surface, and up to 25% of patients can develop peritoneal metastasis (PM) during the course of the disease 1.

Definition of tumor deposits (TD) has been changed over time, since its definition in 1935. They were introduced for the first time in the fifth edition of the American Joint Committee on Cancer (AJCC), published in 1997, at the beginning included in Category T 2. In the seventh edition of the American Joint Committee on Cancer (AJCC), published in 2009, they are redefined and included in category N, creating a new category N1c 3.

In the eighth edition of the American Joint Committee on Cancer in 2018, they were described as “nodules without histological evidence of residual lymph node or identifiable vascular or neural structure” keeping the category N1c. 4.

The origin of TDs remains controversial. The presence of TDs reflects the degree of tumor aggressiveness, possibly through a greater capacity for migrating or infiltrating neighboring mesenchymal tissues. This means that patients with TD+ tumors have a lower survival rate and a higher incidence of recurrence 5.

Between 10 and 22% of patients with colorectal carcinoma have TD in the surgical specimen 6,7. TD have been associated to worse prognosis, especially when they are associated with lymph node involvement 7.

The peritoneal surface is affected in almost a third of patients with CRC. 8 Approximately 7%-8% of cases at the time of primary surgery and in 4%-19% of patients during follow-up develop PM. However, in 25% of patients with metastatic disease, the peritoneal cavity appears to be the only site of spreading 9.

For many years, peritoneal carcinomatosis was considered an end- stage of gastro-intestinal malignancies, with a survival lower to six months. The development of aggressive cytoreductive surgery and HIPEC in addition to perioperative systemic therapy has undergone a significant change 10.

Many risk factors for the development of carcinomatosis have been studied, including studies in which HIPEC is performed prophylactically in locally advanced tumors 11-13, but the relationship between TDs and PM has not been studied thoroughly.

The main objective of this study is to analyze the prognostic value of tumor deposits in non-metastatic colorectal cancer as a risk factor of global recurrence, locoregional recurrence, liver and lung metastasis and recurrence free survival (RFS) and specially as a risk factor for peritoneal metastasis.

## Material and Methods

This was a retrospective observational study. All patients treated for non-metastatic colorectal adenocarcinoma between June 2011 and June 2021 in the General Surgery Department of Hospital Universitario Príncipe de Asturias, Alcala de Henares, Madrid, Spain, were included. The main objective of this study was to analyze the prognostic value of TDs as a risk factor of peritoneal metastasis in patients with non-metastatic CRC. The study adhered to the STROBE guidelines for designing and reporting observational studies. During these years, a prospective computerized database was created by the Coloproctology Unit from which patients were identified. The study was approved by the Ethics Committee of Hospital Príncipe de Asturias (Code: OE 37/2021).

### Patients and data

**Figure 1:**
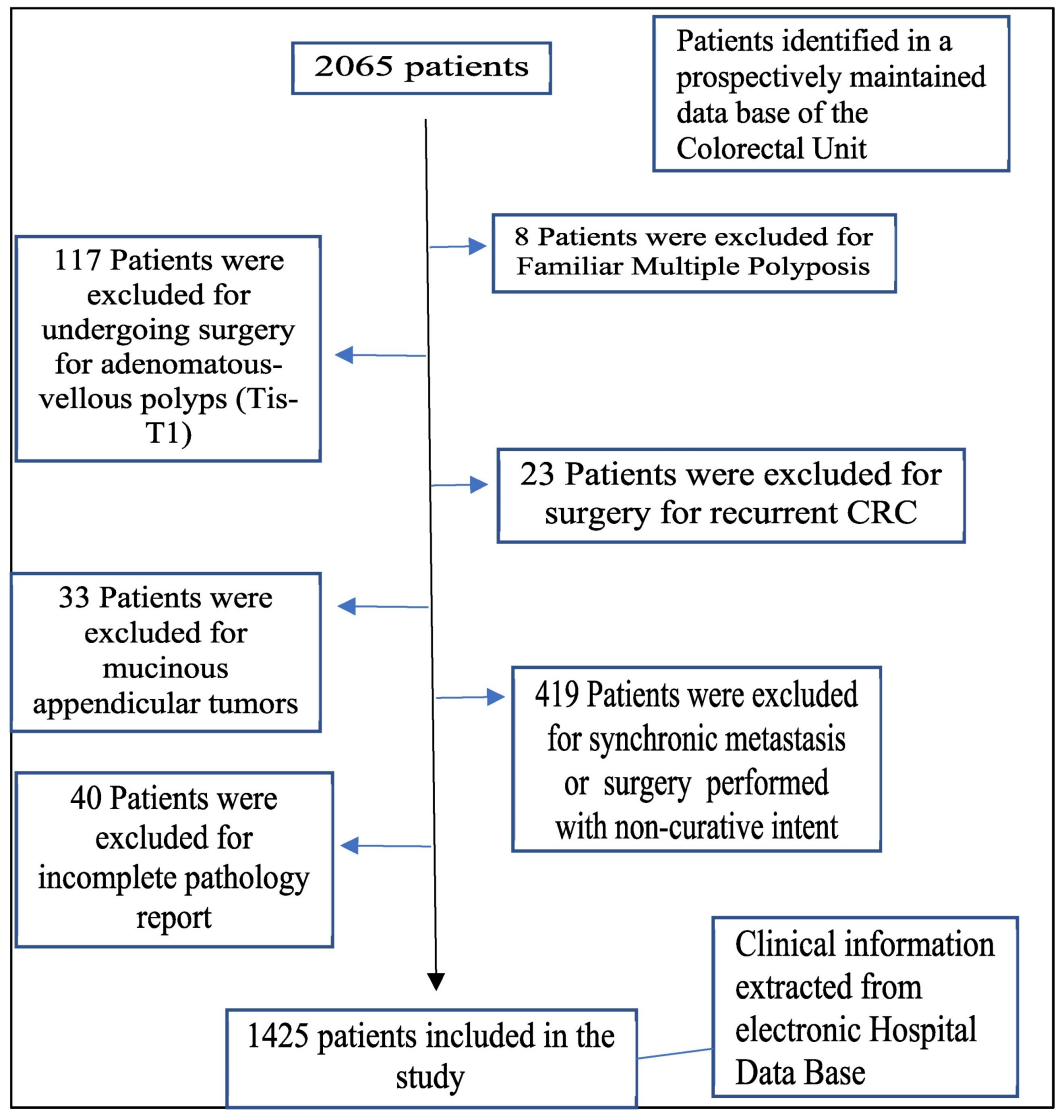
Flowchart detailing the selection of the patients in this study.

The inclusion criteria were patients over 18 years of age, undergoing curative surgery for primary colorectal adenocarcinoma. The exclusion criteria were the following: mucinous appendiceal tumors, adenomatous polyps or tumors *in situ*, familial multiple polyposis, recurrent CRC, palliative surgery, incomplete pathology, and synchronous metastases (Figure 1).

Each patient was evaluated by a multidisciplinary medical committee that determined the possible therapeutic options according to the degree of extension and functional status of the patient. A prospective computerized database was created by the Coloproctology Unit and the clinical data were obtained from the electronic medical records of the hospital.

The predictive variables collected were: demographic information (sex and age), surgical procedures, location of the primary tumor, histopathological information, postoperative morbidity, oncological medical treatment received, and long-term outcome.

Our patient's stage was determined using the eighth edition of the AJCC TNM classification (4). Tumor location was classified as right-sided tumors when the tumor was located in the cecum, ascending colon, hepatic flexure, or transverse colon. Tumors originating from the splenic flexure to the sigmoid colon, including the descending colon, were classified as left-sided tumors. Tumors located between the last 15 cm to the anal verge were defined as rectal tumors. There were no missing data for any of the variables that were included in the analysis.

The post-analysis histopathological report by a gastrointestinal pathologist evaluates the presence of TD, tumor grade, histological type, mucinous component, degree of differentiation, number of lymph nodes examined, number of metastatic lymph nodes, presence of perineural or lymphovascular infiltration. The definition used for TD was that indicated by the eighth edition of the AJCC TNM classification: “macroscopic or microscopic cancer nest, in the lymphatic drainage area of ​​the pericolorectal adipose tissue of a primary carcinoma, discontinuous from the primary and without histological evidence of residual lymph, identifiable vascular or neural node or structure.” (4,5).

Follow-up after surgical treatment was carried out according to current guidelines. During the first 2 years, patients had a physical examination and analytical evaluation every 6 months, and annually thereafter. A computed tomography (CT) scan is performed each year until the fifth year and a colonoscopy in the first and third years after surgery.

### Main outcome measures

The primary outcomes of interest were the development of locoregional recurrence, liver and lung metastasis and PM during the follow up in presence of TDs and Recurrence-free survival (RFS). Overall Survival was estimated from the date of surgery to the date of the last date of follow-up or death and measured in months. Deaths due to colorectal cancer were assumed as deaths to calculate cancer-related survival (CRS), but deaths due to another reason were censored. Recurrence-free survival (RFS) time was defined in months from the date of colorectal surgery to the date of the first recurrence.

### Statistical analysis

The variables were input into a Microsoft Excel 2019 (v.27) (Microsoft, Redmond, WA, USA) spreadsheet. Statistical analysis was performed with SPSS (v.23) (IBM, Armonk, New York, NY, USA).

Clinical and histopathological characteristics among patients with tumors with TDs (TD+) and tumors without TD (TD-) were compared using the χ-squared test. Kaplan‒Meier estimator was use for survival analysis up to 60 months after diagnosis and median survival for each variable included in the present study. To compare survival curves we use the log-rank test.

The effect of each variable on survival was evaluated using Cox proportional-hazard regression. Cox regression models were built using the backward method. Variables included in the adjusted models were those that had p < 0.05 for the outcome of interest in the univariate analysis. These variables were kept in the final model if they were still significant at p < 0.05. The assumption of proportional hazards across different covariates was tested by inspecting the log (-log) plots. The risk of death or recurrence was expressed as the Hazard ratio (HR) with its 95% confidence interval (CI).

## Results

### Patients and characteristics

A total of 1,425 patients met the inclusion criteria. The sex distribution in our sample was 883 (62%) men and 542 (38%) women. The mean age was 68 ± 11 years (range: 69). The mean follow-up was 56 ± 34 months (median: 51). In relation to the ASA classification, 179 patients (12.6%) were ASA I, 769 (54%) ASA II and 477 (33.5%) ASA III.

The tumor was located in the right colon in 471 (33.1%) patients, in the left colon in 576 (40.4%) patients, and in the rectum in 378 (26.5%) patients. Urgent surgery was performed in 189 patients (13.2%), for 123 obstructive tumors (8.6%) and 66 for perforation (4.6%). Overall, 354 (24.8%) patients had TNM stage I tumors, 582 (40.8%) had stage II tumors, and 489 (34.3%) had stage III tumors. The pathological anatomy report showed that the main histologic type was colorectal Adenocarcinoma in 1292 patients (90.7%) and mucinous tumor in 133 patients (9.3%). Poorly differentiated tumors were identified in 109 patients (7.6%), lymphovascular infiltration and perineural infiltration were present in 223 (15.6%) and 203 (14.2%) patients. Adjuvant therapy was administered after primary surgery in 624 patients (43.8%). Table 1 shows the distribution of the clinical and histopathological characteristics of the patients.

### Patient and tumor characteristics categorized by the detection of tumor deposits

**Figure 2:**
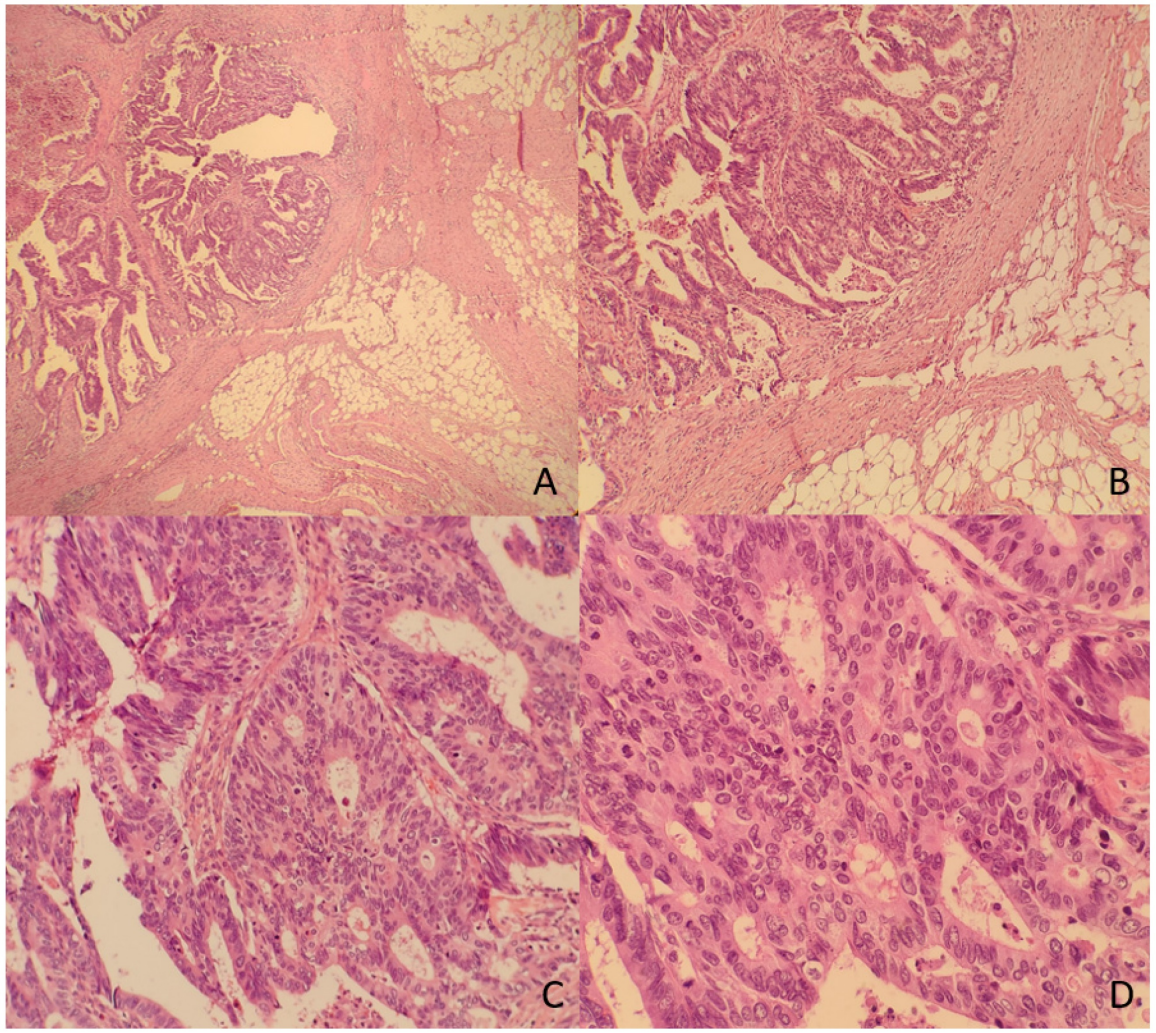
** A.** Panoramic view of a tumor deposit (4x). **B.** A focus of moderately differentiated colorectal adenocarcinoma (G2) with fibroadipose tissue infiltration (10x) is observed. C and D. At higher magnification, tubular and cribriform formations with a proliferation of atypical epithelioid cells with marked pleomorphism and numerous mitoses are identified (20x and 40x).

**Figure 3:**
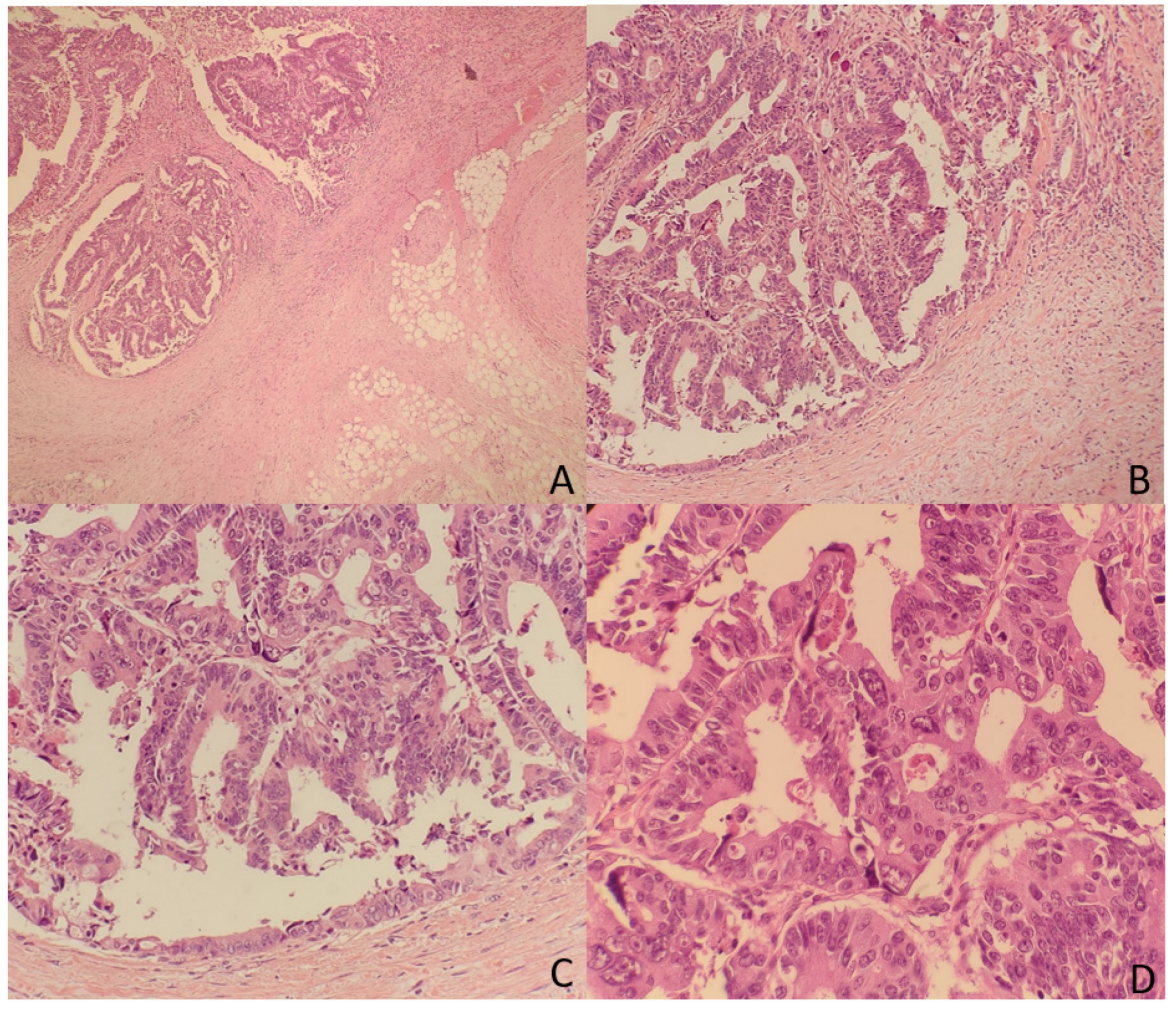
**A.** Panoramic view of a tumor deposit (4x) in relation to fibroadipose tissue without associated lymphoid tissue or infiltrated vascular or neural structures. **B, C, and D.** At higher magnification, a focus of well-differentiated colorectal adenocarcinoma (G1) is observed in relation to desmoplastic fibrous tissue (10x). B and C. The tumor is composed of glandular structures of different sizes with foci of intraglandular tumor necrosis (arrows) (20x and 40x). There were no statistically significant differences in the incidence of TDs by sex, age, ASA, primary tumor location, or histologic type. TDs were associated with higher T stage (0.6% in T1, 2.8% in T2, 13.6% in T3 and 30.6% in T4; p < 0.001), poorly differentiated tumors (25.7 vs. 11.2%; p < 0.001), lymphovascular infiltration (39 vs. 7.4%; p < 0.001), perineural infiltration (37.8 vs. 8.2%; p < 0.001), tumors that presented intestinal obstruction (17.9 vs. 11.8%; p = 0.041), and perforated tumors (30.3 vs. 12.5%; p < 0.001). Among the 176 tumors with TDs, 87 (49.4%) also had lymphovascular infiltration, and 76 (43.2%) also had perineural infiltration. At 60 months of follow-up, 264 patients (18.5%) died due to CRC. Cancer-related survival (CRS) at 5 years was lower in patients with TD (41.2% vs. 83.3%; p < 0.001) (OR: 4.95; 95% CI: 3.830-6.394). (Figure 4)

The TDs rate in our non-metastatic colorectal cancer sample was 12.4%, detected in 176 patients. Table 1 shows patient and tumor characteristics categorized by presence of Tumor Deposits. Figure 2 and 3 show H&E images of two tumor deposits.

### Global recurrence and local recurrence categorized by the presence of TDs

During follow-up, 329 (23.1%) patients experienced any tumor recurrence. Among the 329 patients whose disease recurred, the most common sites of recurrence were liver (n = 153; 10.7%), lung (n = 126; 8.8%), peritoneum (n = 105; 7.4%) and local recurrence (n = 74; 5.2%). The results of the univariate analysis recurrences categorized by presence of TDs and OR are shown in Table 2.

**Figure 5:**
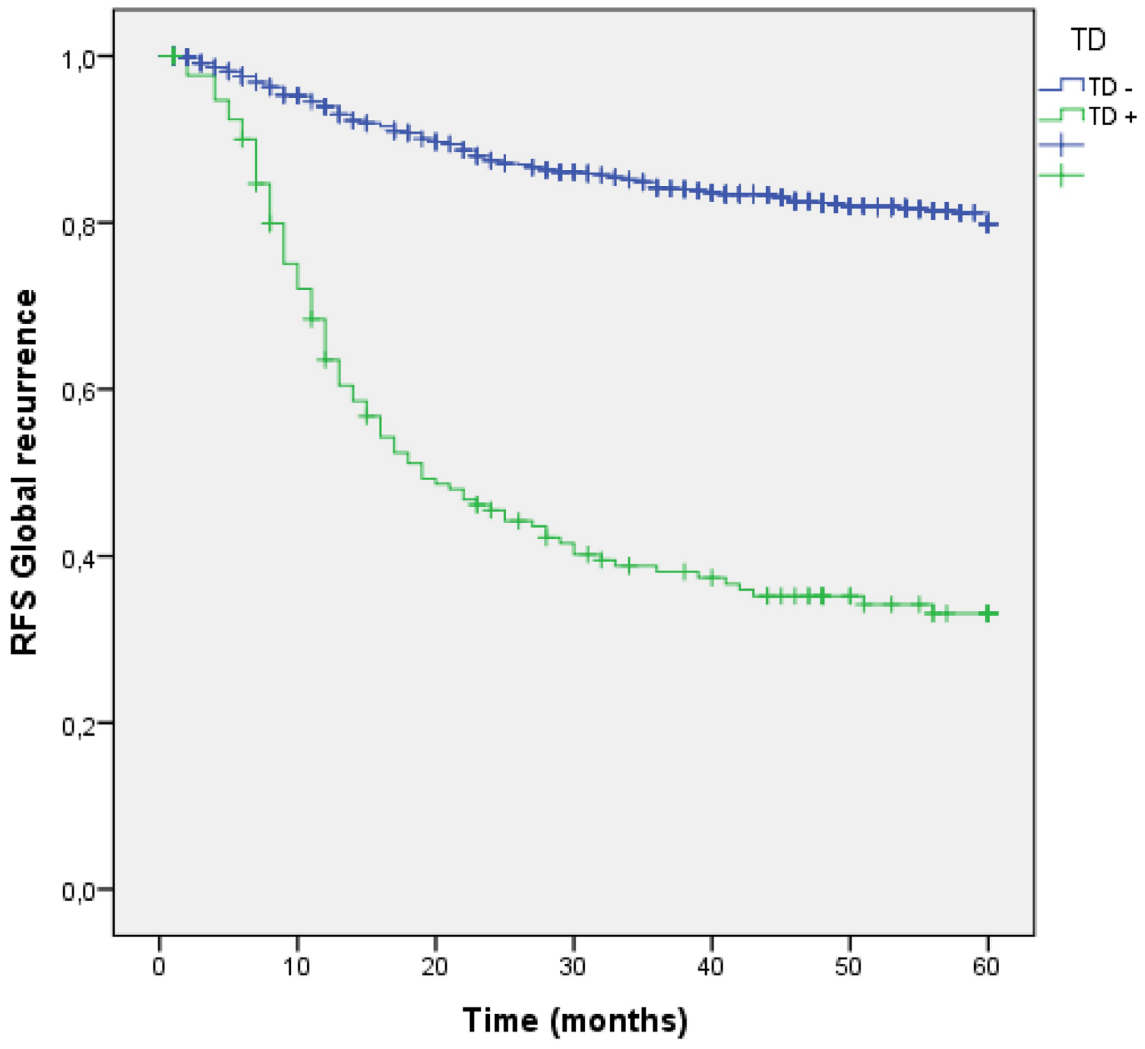
Kaplan-Meier estimates of RFS for the entire cohort according to the presence of Tumor Deposit.

We analyzed the RFS for global recurrence at 60 months in the entire cohort according to the presence or absence TD. The global recurrence rate in patients with TDs was significantly higher than those without TDs (17.8% vs 60.8%; p=0.001). (Figure 5 estimates of RFS for the entire cohort according to the presence of Tumor Deposit).

#### Analysis by presence/absence of LNM and TD

**Figure 6:**
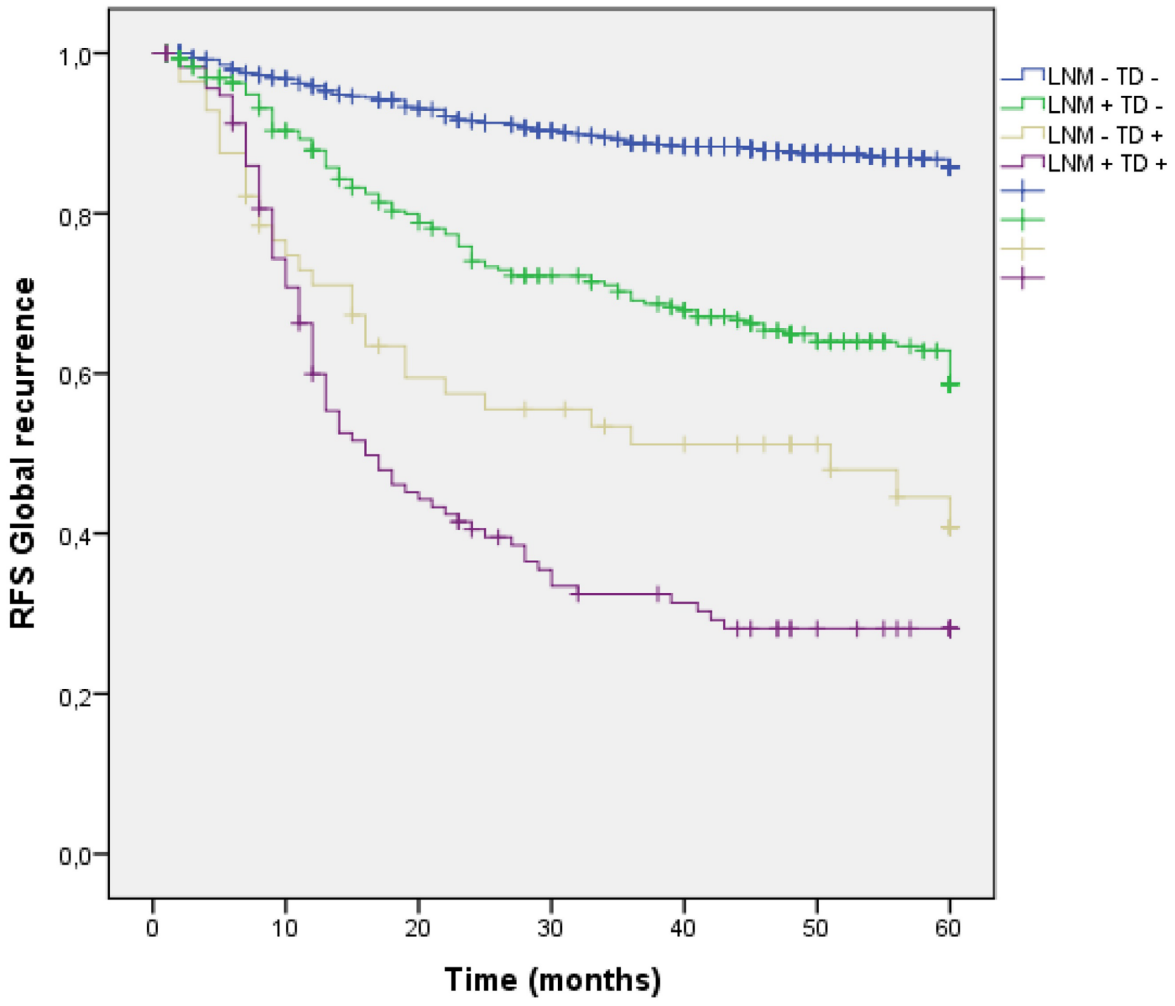
Kaplan-Meier estimates of RFS for the entire cohort according to the presence of Tumor Deposit and LNM.

Four groups of patients were built in the cohort, according to the presence or absence of Lymph Node Metastasis (LNM) or TD. 935 patients (65.6%) had neither LNM nor TD in the pathology report (LNM- TD-), 314 patients (22.1%) had LNM + without TD (LNM + TD-), 59 patients (4.1%) had TD+ without any LNM (LNM- TD+) and finally 117 patients (8.2%) had both (LNM+ TD+). Global recurrence in patients without LNM nor TD (LNM- TD-) was 12.2%, in patients with LNM (LNM + TD-) was 34.1%, in patients with TD without any LNM (LNM- TD+) was 49.2% and in high-risk patients with both risk factors global recurrence was 66.7%;(p<0.001). (Figure 6) Distribution of global, locoregional, liver, lung and peritoneal metastasis by presence of LMN and TD are shown in Table 3.

Local recurrence occurred in 74 patients (5.2%), among the 176 patients with TDs local recurrence rate was higher (4.2% vs 11.9%; p<0.001). Local recurrence occurred in 28 patients (3%) without LNM and TD (LNM- TD-), in 25 patients LNM + TD- (8%), 7 patients with LNM- TD+ (11.9%) and 14 patients with both (12%) (LNM+ TD+); p<0.001) (Table 3).

### Tumor deposits and liver metastasis

**Figure 7:**
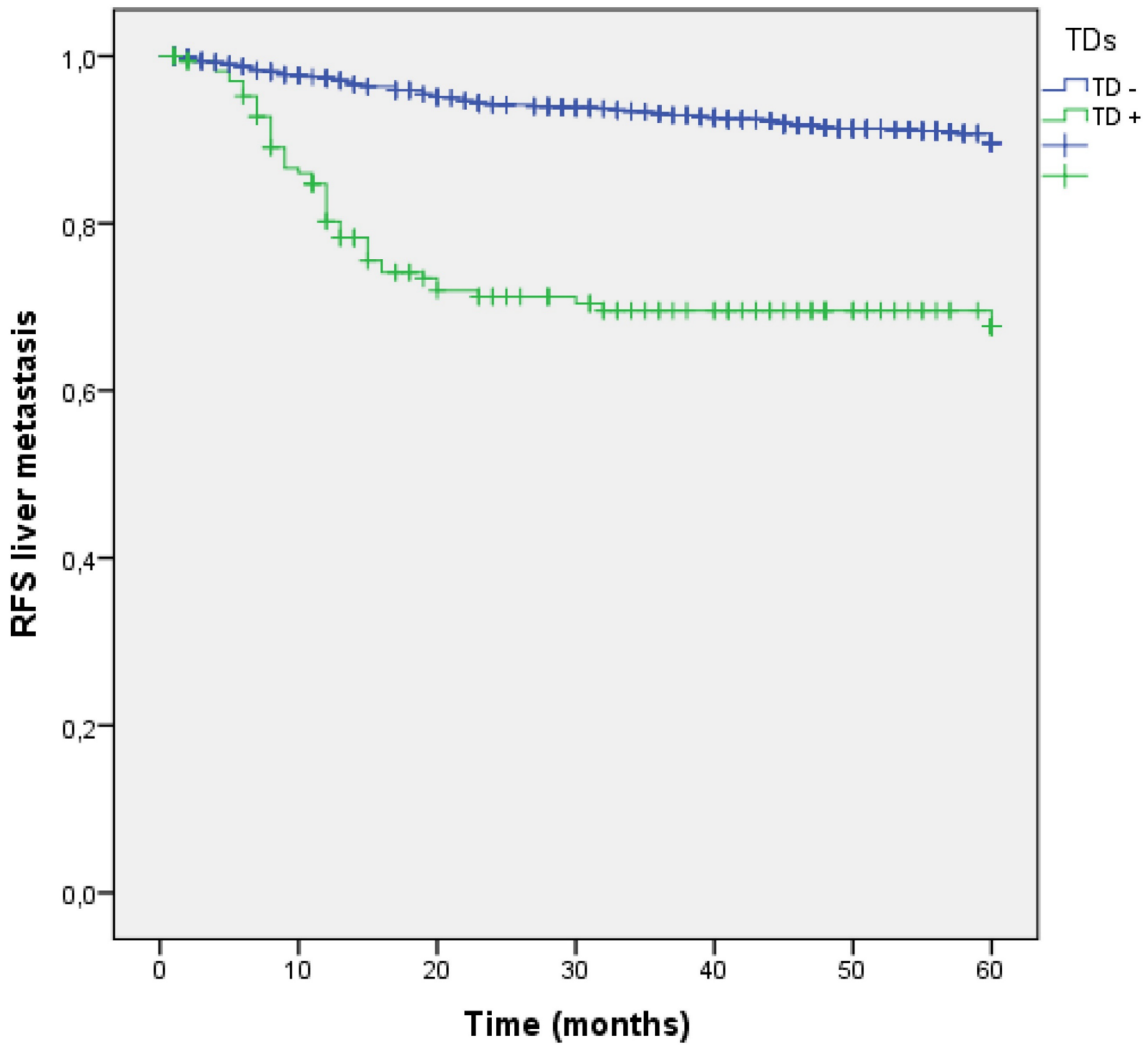
Kaplan-Meier estimates of RFS for liver metastasis according to the presence of Tumor Deposit.

Liver is the most common site of metastasis from CRC, in our study hepatic dissemination occurred in 155 (10.9%) patients during follow-up. Among 176 patients with TDs, 47 (26.7%) suffered liver metastasis, showing the increased risk compared to those without TDs (8.6% vs 26.7%; p<0.001). The negative effect of TD+ on the liver metastasis development had a OR of 4.244 (95% CI: 3.004-5.994). Five years RFS for liver dissemination was higher without TDs (91.4% vs 73.3%; p<0.001) (Figure 7).

#### Analysis by presence/absence of LNM and TD

**Figure 8:**
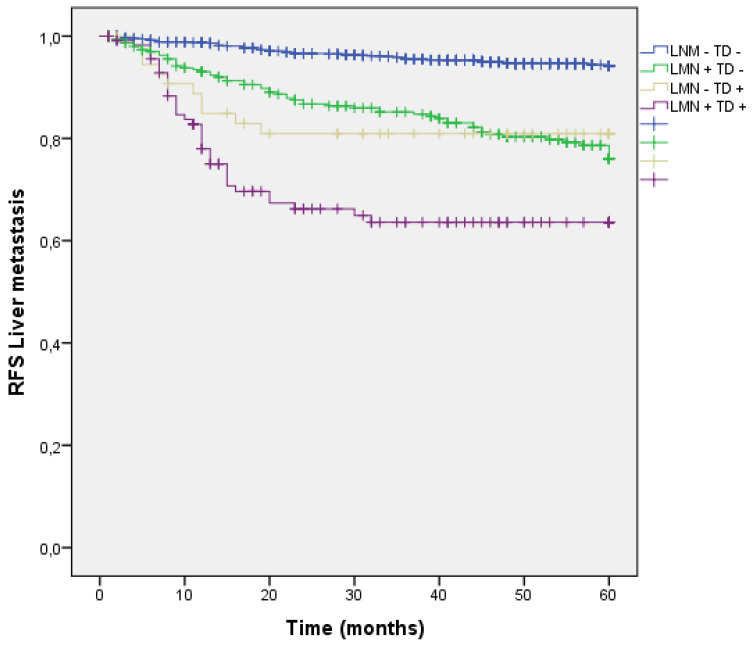
Kaplan-Meier estimates of RFS for liver metastasis according to the presence of Tumor Deposit and LNM.

Liver dissemination was higher in patients with LNM and TD (LNM+ TD+) 37 (31.6%), however patients with LNM + and TD - had a trend toward higher liver metastasis rate than those with TD+ LMN -. (LNM- TD- 4.9%, LNM + TD- 18.8%, LNM- TD+ 16.9%, LNM+ TD+ 31.6%; p<0.001) (Figure 8) (Table 3).

#### Analysis by stage

We evaluated our patients divided by staging. Each TD patient was classified as stage III, so we analyzed only 489 patients belonging to stage III. Among these 489 patients, 176 (36%) had TDs. Patients with TD had a higher rate and higher risk of liver metastasis 48 (27.3%) vs 59 (18.8%); p=0.031 (OR: 1.755 (95% CI: 1.194-2.579). The analysis by stage was summarized in Table 4.

### Tumor deposits and lung metastasis

**Figure 9:**
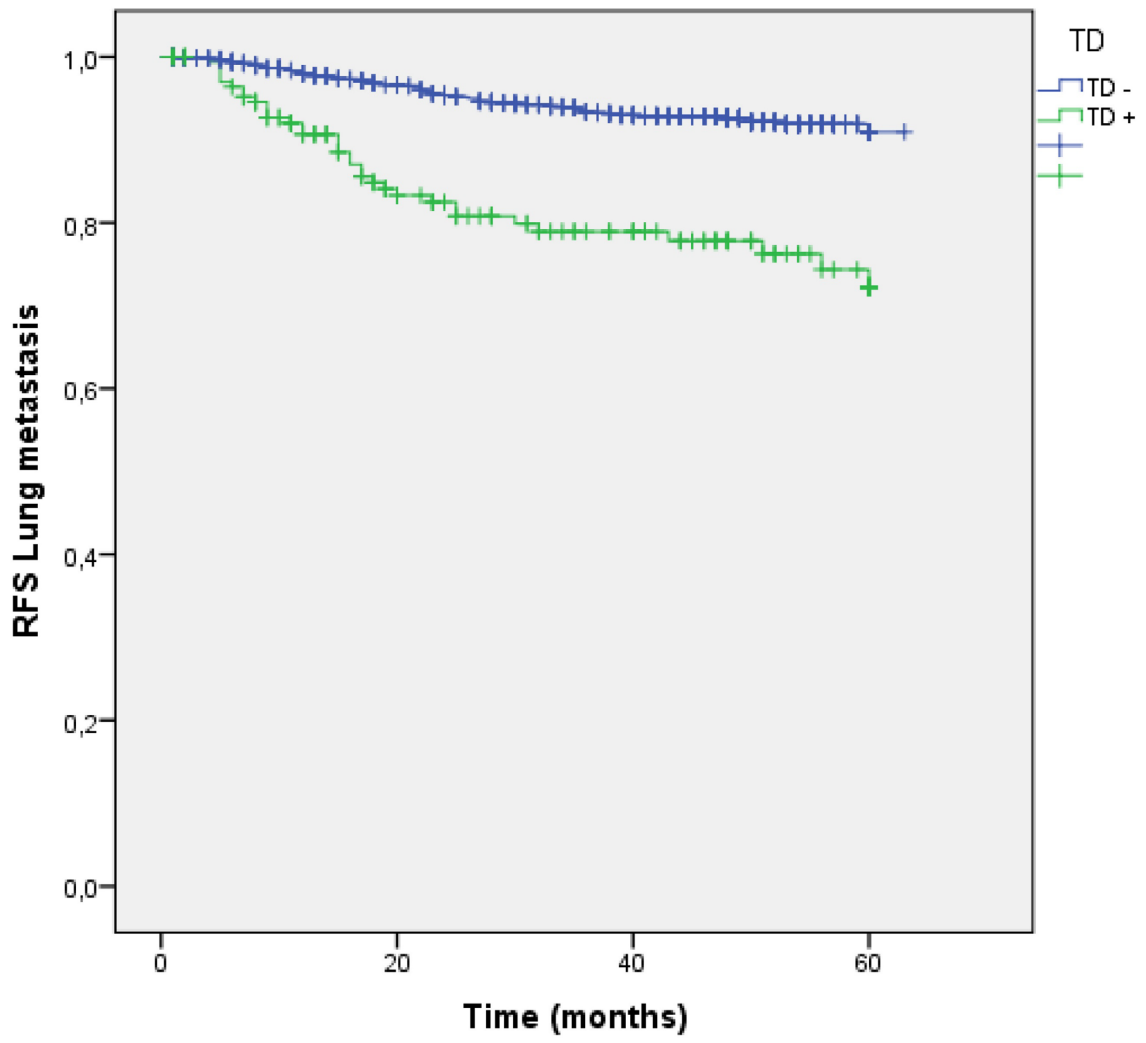
Kaplan-Meier estimates of RFS for lung metastasis according to the presence of Tumor Deposit.

In our sample, 126 (8.8%) patients suffered lung metastasis during follow-up. Patients with TD + had a higher lung metastasis rate (7.4% vs 19.3%; p<0.001) and a greater risk (OR 3.585;95% CI: 2.397-5.362). RFS for lung metastasis is shown in Figure 9.

#### Analysis by presence/absence of LNM and TD

**Figure 10:**
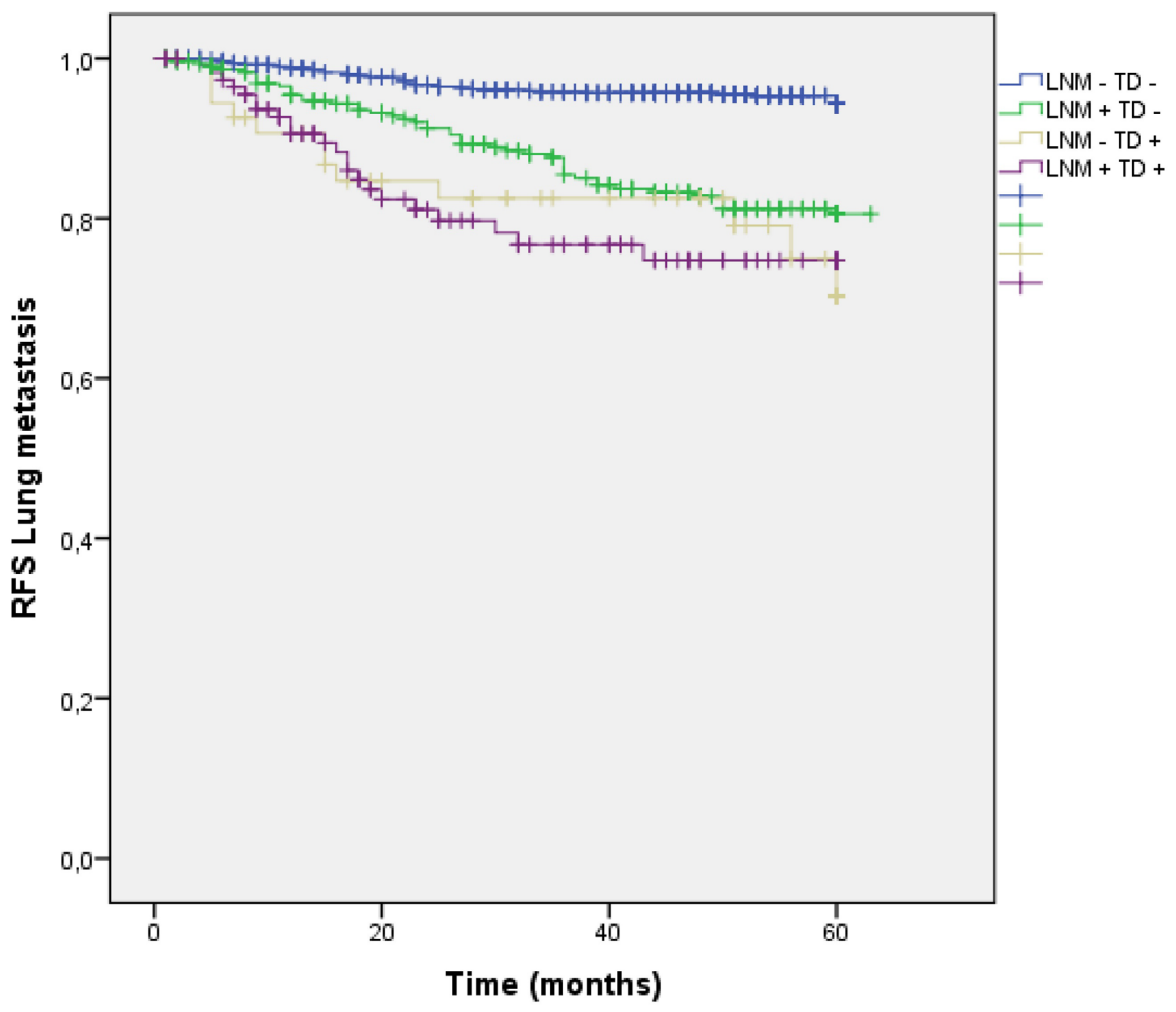
Kaplan-Meier estimates of RFS for lung metastasis according to the presence of Tumor Deposit and LNM.

Distribution by groups based on the presence/absence of LNM and TD shows that patients with TD + and LNM + had a higher rate of lung metastasis. (LNM- TD- 4.3%, LNM + TD- 14.3%, LNM- TD+ 18.6%, LNM+ TD+ 19.7%; p=0.000) (Figure 10).

#### Analysis by stage

Evaluating our Stage III patients, patients with presence of TD also had a higher risk of lung metastasis, without reaching statistical significance 34 (19.3%) vs 47 (15%); p=0.219. OR: 1.612 (95% CI: 1.027-2.531) (Table 4).

### Tumor deposits and Peritoneal Metastasis

**Figure 11:**
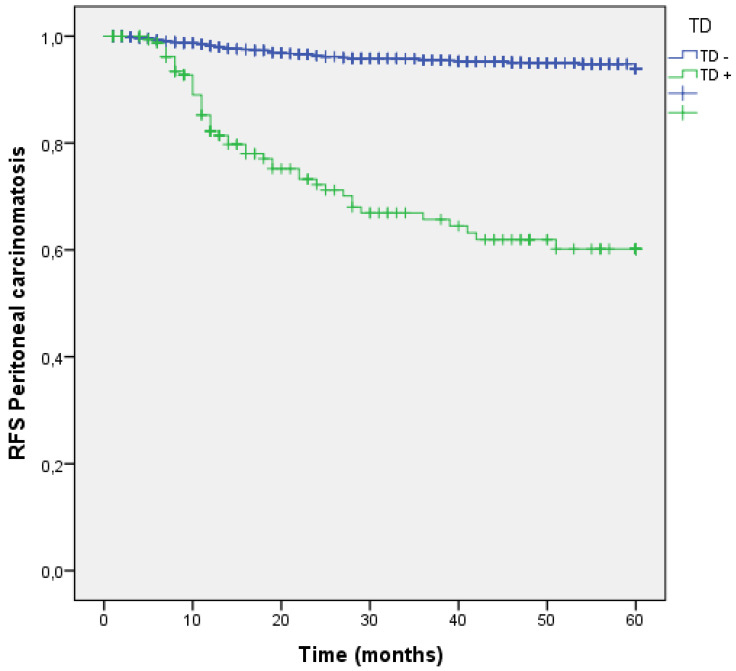
Kaplan-Meier estimates of RFS for peritoneal carcinomatosis according to the presence of Tumor Deposit.

During follow-up, the peritoneal cavity was affected in 105 (7.4%) patients. The group of patients with TDs in the first surgery suffered higher rates of Peritoneal Metastasis (4.7 % vs 26.1 %; p<0.001). Patients with TDs had 7.511 times higher risk to develop PM (OR: 7.511 (95% CI:5.092-11.079). (Table 2). RFS curve for PM at 60 months shows better RFS for those without TD (95.3% vs 73.9%; p<0.001) (Figure 11).

The predictive factors of peritoneal metastasis analyzed using the Cox proportional hazards model are shown in Table 5.

#### Analysis by presence/absence of LNM and TD

**Figure 12:**
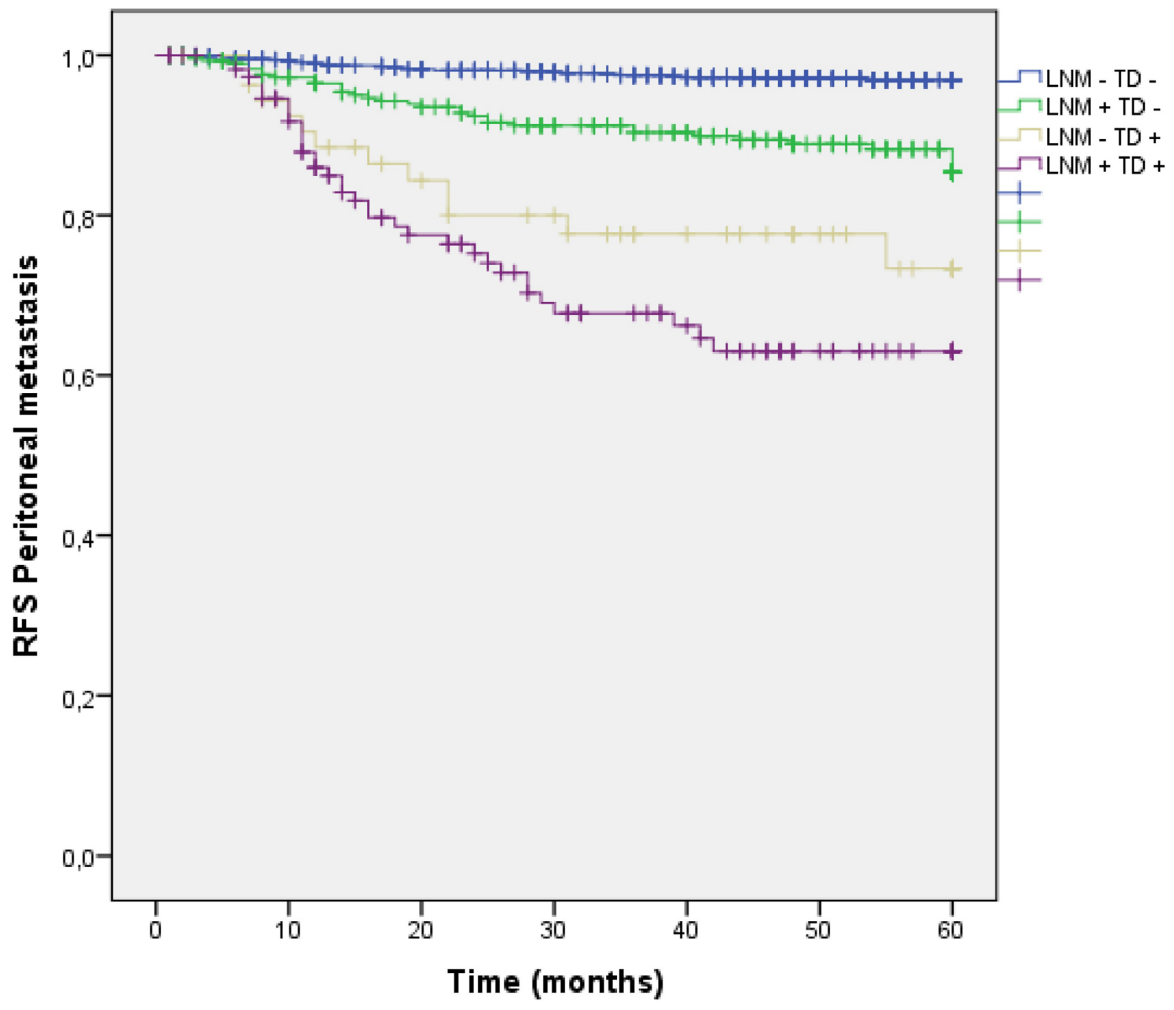
Kaplan-Meier estimates of RFS for Peritoneal metastasis according to the presence of Tumor Deposit and LNM.

Distribution by groups shows that patients with TD + and LNM + had a higher rate of peritoneal metastasis, however patients with TD + without any LMN had higher PM rate than those with LNM + without TD.

(LNM- TD- 2.7%, LNM + TD- 10.8%, LNM- TD+ 20.3%, LNM+ TD+ 29.1%; p<0.001) (Figure 12).

#### Analysis by stage

The main differences in distant metastasis were found in stage III patients with presence of TDs who suffered higher rates and greater risk of PM, 46 (26.1%) vs 34 (10.9%); p < 0.001). OR: 3.075 (95% CI: 1.969-4.803). Recurrences categorized by presence of Tumor Deposit and OR of TD + in Stage III patients are represented in Table 4.

According to our multiple regression analysis, the presence of TDs had a significant adverse effect on PM in stage III. These results are shown in Table 6.

## Discussion

This study analyzed 1,425 patients with non-metastatic colorectal cancer treated at a single institution over a ten-year period. The presence of TDs in CRC is associated with worse prognosis 14, our results confirmed that patients with TDs had lower CSR and RFS.

Although TDs have been known for almost a century, their pathobiological significance is still poorly understood and their origin and mode of spread remain controversial 15. TDs are discontinuous disseminated tumors in the mesocolon/mesorectum in the absence of lymphatic or vascular structures, separated from the primary tumor. Some studies propose that the relationship between LNM and TD suggests that tumor cells spread through lymphatic channels and eventually replace the lymph node. The association between tumor deposits and vascular invasion suggests their spread through venous invasion. However, most support the hypothesis that tumor deposits spread in multiple ways: through lymphatic, venous and perineural invasion, and discontinuous extension of the primary tumor 16.

The impact of TDs on rectal cancer was analyzed in 2022 by Agger *et al.* and show that TDs have a negative impact on prognosis in rectal cancer and local recurrence and distant metastases increased, however they did not discriminate whether there is any different pattern in different distant metastasis sites 17. In 2024, Hakki *et al.* analyzed the association between TD and colon cancer recurrence in 770 stage I-III patients. They found that TD independently predicts recurrence. Patients with LNM and TD have twice the risk of recurrence than patients with only LNM. However, they also did not discriminate whether there is any different pattern at different sites of distant metastasis. 18 Our study shows that patients with TDs had a lower CSR and suffered higher rates of local recurrence, liver and lung metastases but, above all, patients with TDs had a 7.511 times higher risk of developing PM. Furthermore, according to our multiple regression analysis, the presence of TDs was associated with an increased risk of PM. In Stage III patients, the presence of TDs also was an independent factor that increase the risk of distant metastasis; however, the main risk was to develop peritoneal metastasis. LNM and TD are histopathological variables that are associated to worse prognosis, in our results we show that patients with both LNM + and TD + had a higher recurrence rate, especially PM, however patients with TD + without LNM had higher rate of PM than those with LNM + without TD, which may reflect that the presence of TD is an independent prognostic factor for PM.

There are few studies demonstrating the effect of the number of tumor deposits in recurrence, but some authors propose that patients with more than 4 TD have a higher risk of local and distant recurrence compared with those with <4 TD. Bendari *et al.* showed that 14% of patients had more than 4 TD and 61.5% of them presented some metastasis, compared with 27.7% of metastasis in patients with less than 4 TD 19.

Hakki *et al.* showed that the number of TD and the number of LNM have a weak correlation. 91% of patients with TD had 3 or fewer TD, and 46% had a single TD, but they did not look for worse prognosis in patients with more than one TD 18.

The presence of TD reflects the degree of aggressiveness of the tumor, possibly through a greater ability to migrate or infiltrate neighboring mesenchymal tissues. They hypothetically may represent lymph nodes or vascular or nervous structures completely filled with carcinoma 7 or TDs may play a role as a first step of peritoneal dissemination in a biologically very aggressive cancer. Our results are in agreement with the data provided by Khan *et al.* in 2023, that suggests that non-metastatic colorectal cancer patients with TDs might have a higher risk of peritoneal recurrence 20. These results reinforce our previous study in which TDs represents an independent prognostic value and provided complementary information to that provided by LNM and means that patients with TDs tumors have a lower survival rate and a higher incidence of recurrence 5,6,7.

According to molecular markers, some molecular subtypes have shown poor survival and worse prognosis, like the mesenchymal CMS4 subtype 21. Brouwer *et al.* in 2023 concluded that TDs show a more invasive phenotype compared to LNM, based on differences in gene expression, TDs represent mainly the CMS4 phenotype reflecting their more aggressive biology 22. Furthermore, TDs are associated with a transcriptional factor that induces epithelial-mesenchymal transition and inhibits E-cadherin that enhance angio-invasiveness promoting tumor invasion 23.

Chen *et al.* in 2022 constructed a predictive nomogram that identified the high-risk TD-positive patients. They recommend that for the high-risk or medium-risk subgroup, additional chemotherapy and close follow-up may bring benefits for the TD-positive patients 24.

Several studies have analyzed some risk factors for PM, such as perforation, obstruction, locally advanced tumors, LNM, mucinous adenocarcinoma, poor differentiation, elevated serum CEA, elevated serum CA19-9 and positive peritoneal cytology 25. However, despite TDs representing more aggressiveness, usually they are not analyzed as a risk factor of peritoneal metastasis 26. A recent systematic review of risk factors for PM after curative colorectal surgery did not analyze TDs 27. That suggests that TDs are underestimated. We also consider that TDs are underestimated in the current TNM system, mainly in patients who simultaneously present LNM.

We must be aware that TDs have independent prognostic value and provide complementary information. Patients with TDs have worse CSR, RFS and higher recurrence rates, especially PM. In Stage III patients, TDs also was an independent factor that increase the risk of PM. We have to be more aggressive in the treatment and follow-up of patients with TDs, because as we have described, the biology of the tumor is. More prospective studies are needed to determine whether TDs progress to PM.

There are several limitations that must be considered. This is a single institution study over a long period of time (ten years), however we have a large sample size (n=1425). The number of tumor deposits was not analyzed because we do not have this information in the pathological report during the first years.

## Conclusions

In our study, all types of recurrences but especially peritoneal metastases were associated with presence of tumor deposits in patients with non-metastatic colorectal cancer. Patients with TD + without LNM had higher rate of peritoneal metastasis than those with LNM + without TD. The results of our study showed that TD have independent prognostic value and provide complementary information. The presence of TD increases the risk of peritoneal metastasis. Prognostic value of TDs is underestimated in the current TNM system.

## Figures and Tables

**Figure 4:**
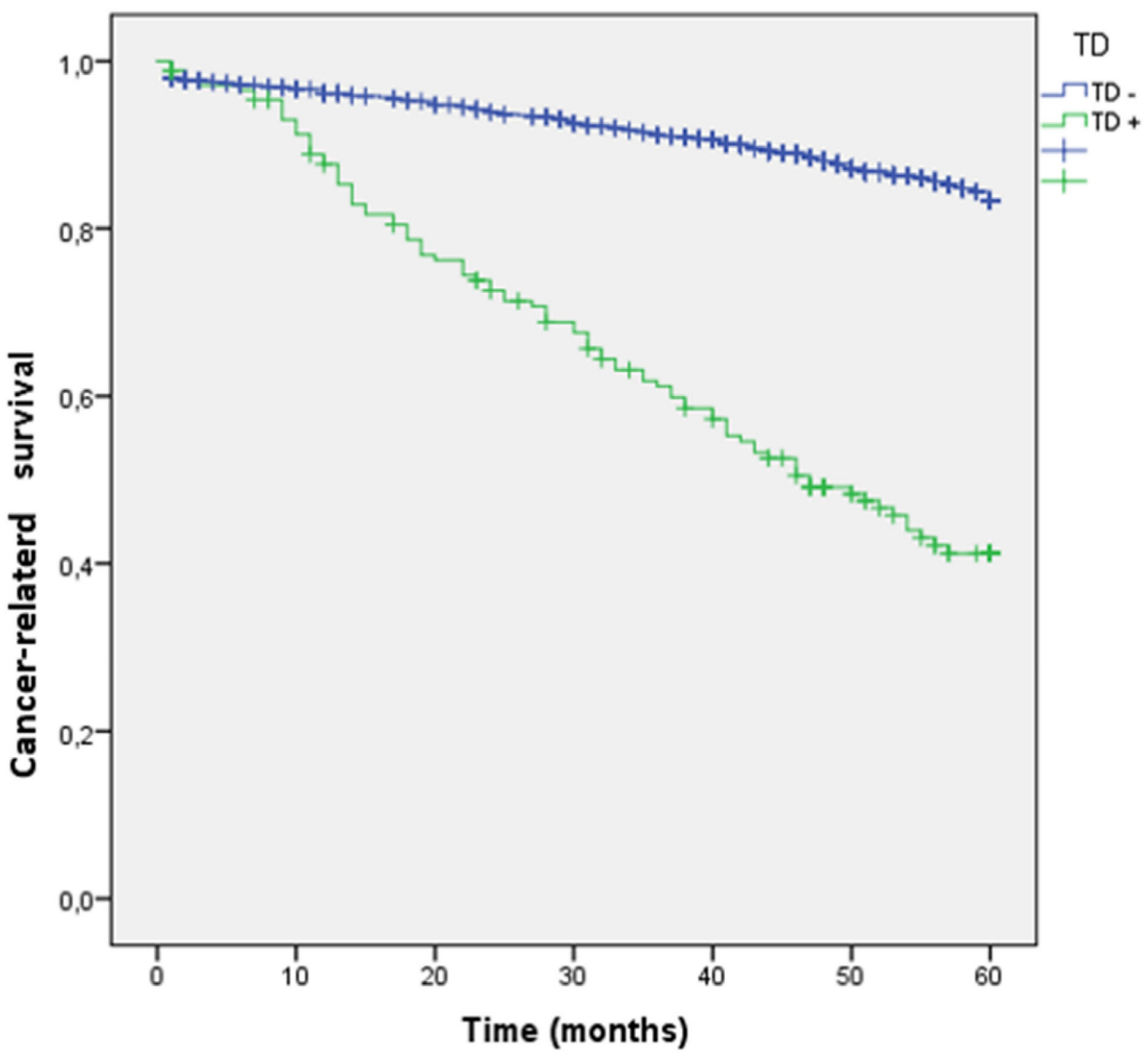
Kaplan-Meier estimates Cancer-related survival (CRS) by presence of Tumor Deposit.

**Table 1:** Patient and tumor characteristics categorized by presence of Tumor Deposit

	Number of patients (n=1425)	Tumor deposit negative n=1249 (87.6%)	Tumor deposit positive n=176 (12.4%)	P value
**Sex**				0.823
Women	542	472 (87.1%)	70 (12.9%)	
Men	883	777 (88%)	106 (12%)	
**Asa**				0.233
I	179	163 (91.1%)	16 (8.9%)	
II	769	675 (87.8%)	94 (12.2%)	
III	477	411 (86.2%)	66(13.8%)	
**Tumor site**				0.915
Right colon	471	414 (87.9%)	57 (12.1%)	
Left colon	576	506 (87.8%)	70 (12.2%)	
Rectum	378	329 (87%)	49 (13%)	
**Age (years)**				0.333
<50	105	94 (89.5%)	11 (10.5%)	
50-69	608	540 (88.6%)	68 (11.4%)	
>69	712	614 (86.2%)	98 (13.7%)	
**T stage**				<0.001
T1	161	160 (99.4)	1 (0.6)	
T2	247	240 (97.2)	7 (2.8)	
T3	844	729 (86.4)	115 (13.6)	
T4	173	120 (69.4%)	53 (30.6%)	
**Lymph node metastasis**				<0.001
0	935	935 (94.1%)	59 (5.9%)	
1-3	380	260 (81.3%)	119 (18.8%)	
>3	110	54 (48.6%)	57 (51.4%)	
**TNM**				<0.001
I	354	354 (100%)	0	
II	582	582 (100%)	0	
III	489	313 (64%)	176 (36%)	
**Tumor grade**				<0.001
Well-moderately differentiated	1316	1168 (88.8%)	148 (11.2%)	
Poorly differentiated	109	81 (74.3%)	28 (25.7%)	
**Lymphovascular infiltration**				<0.001
No	1202	1113 (92.6%)	89 (7.4%)	
Yes	223	136 (61%)	87 (39%)	
**Perineural infiltration**				<0.001
No	1222	1124 (91.8%)	100 (8.2%)	
Yes	203	125 (62.2%)	76 (37.8%)	
**Histologic type**				0.084
Adenocarcinoma	1292	1138 (88.1%)	154 (11.9%)	
Mucinous	133	111 (83.5%)	22 (16.5%)	
**Intestinal obstruction**				0.040
Absent	1302	1148 (88.2%)	154 (11.8%)	
Present	123	101 (82.1%)	22 (17.9%)	
**Tumor perforation**				<0.001
Absent	1359	1203 (88.5%)	156 (12.5%)	
Present	66	46 (69.7%)	20 (30.3%)	
**Postoperative adjuvant chemotherapy**				<0.001
No	801	740 (92.4%)	61 (7.6%)	
Yes	624	509 (81.6%)	115 (18.4%)	

χ2 test was used to calculate the P‑values.

**Table 2:** Recurrences categorized by presence of Tumor Deposit and univariate analysis of risk of recurrence

	Number of patients (n=1425)	Tumor deposit negative n=1249 (87.6%)	Tumor deposit positive n=176 (12.4%)	P value	OR (95% ci) +
**Global recurrence**				<0.001	5.226 (4.137-6.603)
Absent	1096 (76.9%)	1027 (82.2%)	69 (39.2%)		
Present	329 (23.1%)	222 (17.8%)	107 (60.8%)		
**Local recurrence**				<0.001	
Absent	1351 (94.8%)	1196 (95.8%)	155 (88.1%)		
Present	74 (5.2%)	53 (4.2%)	21 (11.9%)		
**Liver**				<0.001	4.244 (3.004-5.994)
Absent	1270 (89.1%)	1141 (91.4%	129 (73.3%)		
Present	155 (10.9%)	108 (8.6%)	47 (26.7%)		
**Lung**				<0.001	3.585 (2.397-5.362)
Absent	1299 (91.2%)	1157 (92.6%)	142 (80.7%)		
Present	126 (8.8%)	92 (7.4%)	34 (19.3%)		
**Peritoneal**				<0.001	7.511 (5.092-11.079)
Absent	1320 (92.6%)	1190 (95.3%)	130 (73.9%)		
Present	105 (7.4%)	59 (4.7%)	46 (26.1%)		

OR=Odds ratio

**Table 3:** Distribution of global, locoregional, liver, lung and peritoneal metastasis by presence of LMN and TD

	LNM- TD- n=935; 65.6%	LNM + TD-) n=314; 22.1%	LNM- TD+ n=59; 4.1%	LNM+ TD+n=117; 8.2%	P value
**Global recurrence**					<0.001
Absent	821 (87.8%)	207 (65.9%)	30 (50.8%)	39 (33.3%)	
Present	114 (12.2%)	107 (34.1%)	29 (49.2%)	78 (66.7%)	
**Local recurrence**					<0.001
Absent	907 (97.2%)	289 (92%)	52 (88.1%)	103 (88%)	
Present	28 (3%)	25 (8%)	7 (11.9%)	14 (12%)	
**Liver**					<0.001
Absent	889 (95.1%)	255 (81.2%)	49 (83.1%)	80 (68.4%)	
Present	46 (4.9%)	59 (18.8%)	10 (16.9%)	37 (31.6%)	
**Lung**					<0.001
Absent	895 (95.7%)	269 (85.7%)	48 (81.4%)	94 (80.3%)	
Present	40 (4.3%)	45 (14.3%)	11 (18.6%)	23 (19.7%)	
**Peritoneal**					<0.001
Absent	910 (97.3%)	280 (89.2%)	47 (79.7%)	83 (70.9%)	
Present	25 (2.7%)	34 (10.8%)	12 (20.3)	34 (29.1%)	

**Table 4:** Recurrences categorized by presence of Tumor Deposit and univariate analysis of risk of recurrence in Stage III patients.

Stage III	Number of patients (n=489)	Tumor deposit negative n=313 (64%)	Tumor deposit positive n=176 (36%)	P value	OR (95% CI) +
**Liver**				0.031	1.755 (1.194-2.579)
Absent	383 (78.3%)	255 (81.5%)	128 (72.7%)		
Present	106 (21.7%)	58 (18.5%)	48 (27.3%)		
**Lung**				0.219	1.612 (1.027-2.531)
Absent	408 (83.4%)	266 (85%)	142 (80.7%)		
Present	81 (16.6%)	47 (15%)	34 (19.3%)		
**Peritoneal**				<0.001	3.075 (1.969-4.803)
Absent	409 (83.6%)	279 (89.1%)	130 (73.9%)		
Present	80 (16.4%)	34 (10.9%)	46 (26.1)		

OR=Odds ratio

**Table 5:** Predictive factors of peritoneal metastasis analyzed using Cox's proportional hazards model

	P value	HR	IC 95%
**Obstruction**	0.071		0.958-2.872
Absent		1	
Present		1.65	
**T stage**	0.000		
T2		1	
T3		8.097	2.782-23.567
T4		2.704	0.966-7.566
**N stage**	0.000		
N0		1	
N1		3.944	1.667-5.202
N2		1.84	1.13-2.996
**Tumor deposit**	0.000		1.753-4.426
Absent		1	
Present		2.785	
**Lymphovascular infiltration**	0.020		1.089-2.758
Absent		1	
Present		1.733	
**Histologic type**	0.034		
Adenocarcinoma		1	
Mucinous		1.705	1.042-2.789

**Table 6:** Predictive factors of peritoneal metastasis analyzed using Cox's proportional hazards model in Stage III patients

	P value	HR	IC 95%
**Obstruction**	0.084		0.931-3.090
Absent		1	
Present		1.696	
**Lymphovascular infiltration**	0.001		1.407-3.470
Absent		1	
Present		2.210	
**Histologic type**	0.001		
Adenocarcinoma		1	
Mucinous		2.546	1.504-4.311
**Tumor deposit**	0.001		1.698-4.246
Absent		1	
Present		2.685	
